# New Books

**Published:** 2008-04

**Air Pollution XVI**

C.A. Brebbia, J.W.S. Longhurst

Billerica, MA:WIT Press, 2008. 600 pp. ISBN: 978-1-84564-127-6, $396

**Biological Monitoring: Theory and Applications**

M.E. Conti, ed.

Billerica, MA:WIT Press, 2008. 256 pp. ISBN: 978-1-84564-002-6, $168

**Chemical Leasing Goes Global: Selling Services Instead of Barrels: A Win–Win Business Model for Environment and Industry**

Thomas Jakl, Petra Schwager, eds.

New York:Springer, 2008. 245 pp. ISBN: 978-3-211-73751-4, $79.95

**Climate Solutions: A Citizen’s Guide**

Peter Barnes

White River Junction, VT:Chelsea Green Publishing, 2008. 120 pp. ISBN: 978-1-6035-8005-2, $9.95

**Climate Variability and Extremes during the Past 100 Years**

S. Brönnimann, J. Luterbacher, T. Ewen, H.F. Diaz, R.S. Stolarski, U. Neu, eds.

New York:Springer, 2008. 364 pp. ISBN: 978-1-4020-6765-5, $199

**Coast-Valley Air Pollution**

G. Latini

Billerica, MA:WIT Press, 2008. 300 pp. ISBN: 978-184564-098-9, $153

**Diagnosis: Mercury: Money, Politics, and Poison**

Jane M. Hightower

Washington, DC:Island Press, 2008. 288 pp. ISBN: 1-59726-395-8, $24.95

**Environment and Health: Protecting our Common Future**

K. Duncan

Billerica, MA:WIT Press, 2008. 192 pp. ISBN: 978-1-84564-130-6, $126

**Environmental Toxicology II**

A Kungolos, C.A. Brebbia, M. Zamorano

Billerica, MA:WIT Press, 2008. 300 pp. ISBN: 978-1-84564-114-6, $190

**Epigenetics**

Jörg Tost, ed.

Norwich, UK:Caister Academic Press, 2008. 404 pp. ISBN: 978-1-904455-23-3, $300

**Extremely Low Frequency Fields**

World Health Organization, Environmental Health Criteria Series, No. 238

Geneva:WHO Press, 2007. 543 pp. ISBN: 978-924-157238-5, $50

**Increasing Capacity for Stewardship of Oceans and Coasts: A Priority for the 21st Century**

Committee on International Capacity-Building for the Protection and Sustainable Use of Oceans and Coasts, National Research Council

Washington, DC:National Academies Press, 2008. 156 pp. ISBN: 978-0-309-11376-2, $39

**Molecular Biology of the Gene, 6th ed.**

James D. Watson, Tania A. Baker, Stephen P. Bell, Alexander Gann, Michael Levine, Richard Losick

Woodbury, NY:Cold Spring Harbor Laboratory Press, 2008. 841 pp. ISBN: 978-080539592-1, $155.60

**Neither Gods Nor Beasts: How Science Is Changing Who We Think We Are**

Elof A. Carlson

Woodbury, NY:Cold Spring Harbor Laboratory Press, 2008 200 pp. ISBN: 978-087969786-0, $29

**Reaching the Poor: Challenges for Child Health in the Western Pacific Region**

WPRO Nonserial Publication

Geneva:WHO Press, 2007. 72 pp. ISBN: 978-929-061246-9, $10

**Smokeless Tobacco and Some Tobacco-specific** ***N*-Nitrosamines IARC Monographs, Vol. 89**

IARC Monographs on the Evaluation of Carcinogenic Risks to Human

Geneva:WHO Press, 2007. 635 pp. ISBN: 978-928-321289-8, $55

**The Hot Topic: What We Can Do About Global Warming**

Gabrielle Walker, David King

London, UK:Bloomsbury, 2008. 288 pp. ISBN: 978-0-15603-318-3, $14

**The Upside of Down**

Thomas Homer-Dixon

Washington, DC:Island Press, 2008. 448 pp. ISBN: 1-59726-065-7, $18.95

**The Law in the Laboratory: A View from Two Benches**

Robert Charrow

Woodbury, NY:Cold Spring Harbor Laboratory Press, 2008. 160 pp. ISBN: 978-0-8796-9778-5, $39

**Where We Stand: A Surprising Look at the Real State of Our Planet**

Seymour Garte

New York:Amacom, 2007. 304 pp. ISBN: 978-0-8144-0910-7, $24.95

